# Symbolic and non-symbolic predictors of number line task in Italian kindergarteners

**DOI:** 10.3389/fpsyg.2023.1137607

**Published:** 2023-05-02

**Authors:** Carla Meloni, Franco Delogu, Rachele Fanari

**Affiliations:** ^1^Department of Pedagogy, Psychology, Philosophy, Faculty of Humanities, University of Cagliari, Cagliari, Italy; ^2^Department of Humanities, Social Sciences and Communication, Lawrence Technological University, Southfield, MI, United States

**Keywords:** number line estimation task, symbolic numerical competence, non-symbolic numerical competence, kindergarteners, mathematics, number processing

## Abstract

The number line estimation task (NLE) is often used as a predictor for broader measures of mathematical achievement. In spite of its popularity, it is still not clear whether the task is based on symbolic or non-symbolic numerical competence. In particular, there is only a very limited amount of studies investigating the relationship between NLE performance and symbolic vs. non-symbolic math skills in children who have not yet begun formal schooling. This study investigates the strength of the association between NLE performance and symbolic and non-symbolic tasks in young kindergarteners. Ninety two 5-year-old children completed the NLE task (range 0–100) and a battery of early numerical competence tests including symbolic-lexical tasks, symbolic semantic tasks, and non-symbolic semantic tasks. The relationship between symbolic and non-symbolic early numerical competence and NLE performance was analyzed using a regression model based on the Bayesian Information Criterion (BIC). Results show that only symbolic semantic tasks are significant predictors of NLE performance. These results suggest that symbolic numerical knowledge is involved in number line processing among young children, whilst non-symbolic knowledge is not. This finding brings new data to the debate on the relationship between non-symbolic numeral knowledge and symbolic number processing and supports the evidence of a primary role of symbolic number processing already in young kindergarteners.

## 1. Introduction

The main purpose of the present study is to investigate whether and how early symbolic and non-symbolic numerical competencies differently contribute to kindergarteners' numerical representation as measured by the number line estimation task (NLE) (Siegler and Opfer, 2003).

The dynamics between symbolic and non-symbolic systems during the early development of numerical representation is still debated (see, for instance, Carey, 2001, 2004; Siegler, 2016).

Dehaene (1992) proposed the mapping hypothesis, in which a core representation of quantities based on an approximate numerical magnitude system constitutes the cognitive foundation of mathematical cognition. According to this theory, when a child learns the number words in the counting routine, the number symbols take their meaning from the connections they establish with the Approximate Number System, ANS, represented through a mental number line (Dehaene, 1992; Gallistel and Gelman, 1992). Importantly, the mapping hypothesis also claims that the more mature symbolic abilities are based on previous non-symbolic representations (Barth et al., 2005; Mundy and Gilmore, 2009). The mapping hypothesis is supported by evidence of children with number processing difficulties or developmental dyscalculia who show problems with discriminating both non-symbolic and symbolic numerosities (e.g., Landerl et al., 2009; Mussolin et al., 2010; Mazzocco et al., 2011a). Additional support for the mapping hypothesis comes from studies investigating how number acuity (i.e., the ability to discriminate non-symbolic quantities) is associated with math achievement in children and young teenagers (Halberda et al., 2008; Gilmore et al., 2010; Mazzocco et al., 2011b). Finally, the distance effect, a phenomenon by which more distant magnitudes (e.g., 2 and 7) are easier to differentiate than neighboring magnitudes (e.g., 8 and 9) (for a review, see Gallistel and Gelman, 2005) has been observed in both symbolic and non-symbolic comparison tasks, consistently with the mapping hypothesis. However, it should be noted that comparing dot stimuli (non-symbolic) takes significantly longer than comparing digits (symbolic) and that only the digit distance effect appears to correlate with mathematical skills (Rousselle and Noël, 2007; Holloway and Ansari, 2009; Lonnemann et al., 2011).

Several studies, on the other hand, suggest a distinct systems' hypothesis, in which non-symbolic and symbolic magnitude representations follow different developmental trajectories and, consequently, that the link between non-symbolic and symbolic numeral knowledge is weak. For example, a number of studies have shown that non-symbolic magnitude discrimination is only weakly related to overall math achievement (see Chen and Li, 2014; Fazio et al., 2014, and Schneider et al., 2017 for meta-analysis). Moreover, other studies have found that training children in non-symbolic numerical magnitude discrimination does not help the knowledge of symbolic magnitudes (see Wilson et al., 2006; De Smedt et al., 2013 for review). Also in contrast with the mapping hypothesis are a number of studies indicating dissociated impacts of the exact-symbolic and the approximate-non-symbolic systems on math development. These studies suggest that symbolic and non-symbolic systems are separate but coexisting (Holloway and Ansari, 2009; Noël and Rousselle, 2011; Sasanguie et al., 2017). For example, Sasanguie and colleagues in a study with young adults, have found that non-symbolic and symbolic representations have completely independent effects on mathematical performance (Sasanguie et al., 2017) and a similar dissociation has been also found in children: Sasanguie et al. (2014) found that the performance in a dots comparison task (non-symbolic) does not predict the accuracy in a number comparison (symbolic) task performed 6 months later. According to Xenidou-Dervou et al. (2015) symbolic arithmetic processing is modulated by age and education more than non-symbolic arithmetic processing, suggesting that non-symbolic and symbolic arithmetic follow different developmental trajectories.

An elective method to study the mental representation of numbers is the number line estimation (NLE) task. In the classic version of the task, a participant is presented with a number and asked to locate its position on a physical number line representing a certain numerical range (Siegler and Opfer, 2003).

The NLE task is an assessment tool currently adopted by many researchers as a measure of the central components of mathematical reasoning (Schneider et al., 2018). While the NLE task use is widespread in the estimation of math skills, there are different accounts on the nature of the components that NLE is able to assess: (a) NLE is based on the representation of numerical magnitudes (Siegler and Opfer, 2003); (b) NLE mainly requires proportional reasoning (Barth and Paladino, 2011); (c) NLE might also be sensitive to spatial skills, in addition or in alternative to magnitude or proportional processing (Gunderson et al., 2012). Schneider underlines that these accounts do not exclude each other because several studies found that participants in a sample differ in their estimation patterns as well as in their estimation strategies.

NLE performance has been widely used to measure how children represent numbers and how this representation changes over time (Siegler and Booth, 2004; Booth and Siegler, 2006, 2008; Berteletti et al., 2010). Results show that NLE performance is strongly associated with mathematical skills, a correlation that seems to be stronger during elementary school (see Schneider et al., 2018 for meta-analysis; Nuraydin et al., 2023).

The NLE task is considered a reliable instrument to estimate the representation of the mental number line within the Triple Code theoretical framework (Dehaene, 1992). According to Dehaene, the mental number line is a crucial element of our innate ability to represent numerosity and it deploys the analogical (non-verbal and non-symbolic) code to understand quantities. Within this framework, the NLE task would reflect the innate mental number line used by children to represent numerical magnitude, asking subjects to translate between numerical and spatial representations without assuming knowledge of specific measurement units.

While the theoretical framework of the NLE task lays on an analogical, non-symbolic representation, the symbolic vs. non-symbolic nature of the NLE task is still debated (Sasanguie et al., 2013; Daker and Lyons, 2018; Hawes et al., 2019; Yuan et al., 2020). Specifically, if NLE performance at a younger age depends on an approximate-analogical representation of magnitude, we should expect a strong association of NLE performance with non-symbolic knowledge. However, this relationship is not always found, especially in young children.

There are few studies that directly compared symbolic and non-symbolic numerical representations in children using the NLE task. The results of these studies are more in line with a distinct systems hypothesis than with the mapping one. For instance, Sasanguie et al. (2013) found a significant correlation between NLE performance and a symbolic comparison task, but no correlation with non-symbolic tasks in 6- to 8-years-old children. Daker and Lyons correlated 7-year-old children's performance in symbolic and non-symbolic comparison tasks with 0–100 NLE performance, finding that only the symbolic task was closely related to the NLE task (Daker and Lyons, 2018). Therefore, although the NLE task has often been used as an index of analogical representation, the abovementioned findings highlight the symbolic nature of this task in lower elementary school children. Concerning kindergarteners, there are even fewer studies investigating the symbolic/non-symbolic nature of the NLE task. Hawes et al. (2019) have found that performances in a symbolic comparison task, but not in a non-symbolic one, were significantly correlated with kindergarten children's performance in the NLE task. The authors also found that both the symbolic comparison task and the NLE task are good predictors of mathematics skills, while the non-symbolic comparison task is not.

van‘t Noordende et al. (2020) conducted the only work that directly investigated the symbolic and non-symbolic skills involved in the NLE task. Specifically, they investigated the predictive power of non-symbolic (dots comparison and non-symbolic NLE task) and symbolic (enumeration) tasks at T1 (age 3;6) on symbolic NLE performance at T2 (age 5). They found that non-symbolic number line estimation at 3;6 years predicts symbolic number line estimation at age 5. In contrast, the non-symbolic dot comparison task does not predict symbolic NLE performance at age 5.

Our work aims to clarify the nature of numerical representation systems involved in solving the NLE task in young children and to acquire additional evidence on the relationship between non-symbolic and symbolic number processing at an early stage of numerical acquisition.

The present study examines the relationship between kindergarteners' NLE performance and their symbolic and non-symbolic number knowledge. Operatively, we measured the predictive power of symbolic semantic tasks, symbolic lexical tasks, and non-symbolic semantic tasks on NLE performance.

As the task is symbolic in nature, we fully expect to find that symbolic skills influence the performance of the NLE task. According to the mapping hypothesis, we could expect that both symbolic and non-symbolic abilities predict NLE performance. In particular, considering the young age of the children and the accounts of interconnectedness of symbolic and non-symbolic systems (Dehaene, 1992) in the emergence of mathematical skills, we can expect the involvement of non-symbolic skills in the NLE task in kindergartners.

If we found an influence of non-symbolic abilities on NLE, the results would support the hypothesis that the processing of symbolic quantities is still, at least partially, based on the analogical system in this early stage of math development. Instead, if the non-symbolic is not involved, and only the symbolic ability predicts NLE performance, we could argue that the NLE task can be performed without an involvement of the non-symbolic system already at this young age. This finding would be in support of the view that non-symbolic and symbolic magnitude systems follow different developmental trajectories already in preschool age (i.e. Sasanguie et al., 2014).

## 2. Materials and methods

### 2.1. Participants

Ninety-two typically developing children were recruited from a public kindergarten in the city of Cagliari, Italy. The participants' average age was 67.79 months (SD = 3.5), 44 females.

All the children were native Italian speakers. Both the school and the children's parents agreed to let the students take part in the research study and signed an informed consent form. The local ethics committee granted its approval for the study. The socioeconomic status of the sample as measured by the Family Affluence Scale (Boyce et al., 2006) was middle class (mea*n* = 4.7, SD = 1.67).

### 2.2. Procedure

The children were individually tested in a single session by an experienced psychologist. Sessions took place in a school quiet room, from 8:30 a.m. to 11:45 a.m. during school days. Each session lasted about 20 min. Each participant completed a socioeconomic status questionnaire, a general intelligence test, a 0–100 NLE task, two symbolic-lexical numerical tasks, two symbolic-semantic numerical tasks, and two non-symbolic numerical tasks.

All children completed the socioeconomic questionnaire, the general intelligence test, and finally the 0–100 NLE task in this fixed order. Afterward, the additional numerical tasks were administered in a counterbalanced order, with half of the subjects doing first the symbolic tasks and then the non-symbolic tasks and half of the subjects completing the numerical tasks in the reverse order.

### 2.3. Materials

#### 2.3.1. Socioeconomic status

Children's socioeconomic status was assessed with the Family Affluence Scale (FAS) (Boyce et al., 2006). Specifically, the FAS is a four items questionnaire that asks participants questions about things they are likely to know about their family (car, bedrooms, vacations, computers) with a score ranging between 0 and 9: FAS low score (0,1,2) indicates low affluence, FAS medium score (3,4,5) indicates middle affluence, and FAS high score (6,7,8,9) indicates high affluence.

#### 2.3.2. General intelligence

In order to control for the influence of general intelligence on NLE performance, we assessed children's IQ using the Italian standardization of the Colored Progressive Matrices test (Belacchi et al., 2008).

#### 2.3.3. Number line estimation task

A computerized version of 0–100 NLE task was administered using the software PyNLT (Massidda et al., 2015). In each trial, children were shown an Arabic digit and asked to click with the mouse on a 25-cm horizontal line, marked 0 on the left end and 100 on the right end to indicate where the target number should be placed. Each target number (stimuli: 2 – 3 – 4 – 6 – 18 – 25 – 48 – 67 – 71 – 86, as in Siegler and Opfer, 2003) appeared twice in random order. All stimuli were shown on a laptop screen (14 inches) placed about 40 cm. from the participant. Before administering the NLE task, to verify whether the children were able to understand the 0–100 version of the NLE task and the magnitude of two-digit numbers, we used, and expanded, the method used in many previous studies. Specifically, consistently with Siegler and Opfer, 2003; Berteletti et al., 2010 and Slusser et al., 2013, in a training phase, we asked children to locate the position of “50” in a line delimited by 0 and 100 at the extremes. All children indicated the 50 to be located in the middle of the line. In addition, we also asked children to indicate the position of the four numbers “8”, “17”, “61”, and “82” in a new line clean from any reference or landmarks except for the “0” and the “100”. All 92 children in our sample were able to position the “8” and the “17” in the first half of the line, and the “61” and the “82” in the second half of the line. We interpreted the fact that all children were able to correctly locate the position of the numbers smaller than “50” in the first half and the numbers bigger than “50” in the second half as evidence that they understood the task and that they were also able to understand the value of large two-digit numbers. Such tests not only assessed children's comprehension of the task and the magnitudes, but also provided an opportunity for them to familiarize with the NLE testing environment.

NLE task accuracy was assessed by calculating the percentage of absolute error (PAE) (Siegler and Booth, 2004) according to the following formula: [(Estimate—Target Quantity) / Scale of Estimate] ^*^100. For example, if a child was asked to locate “18” on the 0–100 number line, and placed the mark at the location that corresponded to “10,” the percentage of absolute error would be 8% [(10–18)/100]^*^100.

#### 2.3.4. Symbolic and non-symbolic early number skills

Children's early number skills were evaluated through a selection of tasks from the “Battery for Numerical Intelligence from 4 to 6 years of age (BIN 4–6)” (Molin et al., 2007), a standardized test battery that provides early math normative data for Italian children from 4 to 6 years of age.

To control for the level of children's knowledge of the Arabic numerals (numerical symbols), we included two lexical symbolic numerical knowledge: a Number Recognition (NR) task to evaluate digit comprehension and an Arabic Number Reading (ANR) task to evaluate digit production. The NR test includes 9 trials. In each trial, the child is shown 3 numbers in a 1–9 range printed on a card in a non-ordinal fashion. Then the experimenter pronounces one of the three printed numbers and the child is asked to select the spoken number among the 3 alternatives. In the ANR test, children completed 9 trials in which they read Arabic digits ranging from 1 to 9 and presented in a random order. In addition, semantic number knowledge was assessed through an Arabic Digit Comparison (ADC) task in which children completed 11 trials in which they were asked to determine which of two Arabic digits in the range 1–9 was larger; the trials were presented in random order and the distances between numbers varied from 1 to 6. Moreover, semantic number knowledge was assessed even through the Arabic Digits linear Order (ADO) task (children were asked to order by magnitude the numbers written on five cards, range 1–5) which evaluated children's ability to order Arabic digits. We included two semantic tasks because they focus on different aspects (comparing and ordering) of the same construct. To assess non-symbolic numerical knowledge, children were given a dots comparison (DC) task (children were shown two sets of dots with different magnitudes and were asked “Which is more?”) made by 10 trials, with the distances between dots quantities ranging from 1 to 5. In the dot comparison task, children are asked to compare the magnitude of two sets of dots. To make sure that children made a decision based on numerosity and not on other stimulus features like the size of the dots, the procedure includes a) items in which both sets have dots of the same size, b) items in which the size of the dots in the first set is bigger than the size of the dots in the second set and c) items in which the size of the dots in the first set is smaller than the size of the dots in the second set.

Moreover, to assess non-symbolic early numerical knowledge, children were given a task in which different object cards (balloons of various sizes) had to be ordered from smallest to largest (Objects Linear Order, OLO). OLO is a task commonly used to assess magnitude and seriation ability, both considered components of early number competence (e.g., Tobia et al., 2016). The task requires reasoning about size differences and their relationships in much the same way as reasoning about relationships between numbers and their magnitudes. Even in this case, we used two non-symbolic tasks to distinguish two different aspects (comparing and ordering) of the same construct.

For all the numerical tasks, the number of correct answers was used as an index of accuracy.

## 3. Results

Descriptive statistics for all the measures are reported in Table 1.

**Table 1:** Descriptive statistics of all variables (*n* = 92).

**Task type**	**Task name**	**Index**	**Mean**	**SD**	**Min**	**Max**
General intelligence IQ	Colored progressive matrices	IQ	98.59	14.87	70	130
Socioeconomic status	Family affluence scale	FAS	4.71	1.67	1	9
Symbolic task	Number line task 0–100 (0–100 NLT)	Percentage of absolute error (PAE)	24.30	10.25	6.77	57.71
	Lexical tasks	Number recognition (NR)	8.76	0.83	3	9
		Arabic numbers reading (ANR)	8.49	1.24	1	9
	Semantic tasks	Arabic digits comparison (ADC)	10.53	1.11	5	11
		Arabic digits linear order (ADO)	3.73	2.09	0	5
Non-symbolic task	Semantic tasks	Dots comparison (DC)	9.61	0.57	8	10
		Objects linear order (OLO)	4.58	2.49	0	7

We initially examined the relations between symbolic and non-symbolic tasks and 0–100 NLE performance (PAE) using the correlation index Pearson's r (see Table 2). Our analysis shows a significant negative correlation between PAE and the symbolic tasks ADC and ADO, indicating that the better the children are at comparing and ordering the magnitude of Arabic digits, the fewer errors they make on the NLE task. Furthermore, our analysis shows a significant negative correlation between PAE and lexical tasks (NR and ANR) indicating that fewer errors in the NLE task are associated with greater accuracy in the lexical tasks. As expected, socioeconomic status (FAS) significantly correlates with the NLE task, indicating that lower errors in the NLE task are associated with a higher FAS. No other significant correlations were found between the NLE task and any other measures in the analysis.

**Table 2:** Pearson's correlation between all variables, (*N* = 92).

	**1**	**2**	**3**	**4**	**5**	**6**	**7**	**8**	**9**	**10**
1. PAE 100	1.00									
2. NR	−0.229^*^	1.00								
3. ANR	−0.358^***^	0.689^***^	1.00							
4. ADC	−0.440^***^	0.377^***^	0.611^***^	1.00						
5. ADO	−0.362^***^	0.165	0.284^**^	0.256^*^	1.00					
6. DC	−0.203	0.124	0.271^**^	0.192	0.259^*^	1.00				
7. OLO	−0.109	0.121	0.231^*^	0.237^*^	0.352^***^	0.206^*^	1.00			
8. AGE	−0.068	−0.034	0.127	0.047	0.014	0.255^*^	0.187	1.00		
9. IQ	−0.142	0.168	0.305^**^	0.311^**^	0.323^**^	0.192	0.447^***^	0.098	1.00	
10. FAS	−0.253^*^	0.123	0.238^*^	0.138	0.200	0.291^**^	0.281^**^	0.289^**^	0.204	1.00

*** p < 0.001;

** p < 0.01;

* p < 0.05.

PAE, percentage of absolute error 0–100 NLT; NR, numbers recognition; ANR, Arabic numbers reading; ADC, comparison of Arabic digits; ADO, Arabic digits linear order; DC, dots comparison; OLO, objects linear order; AGE, age in months; IQ, general intelligence; FAS, children's socioeconomic status.

To control for gender effects in the NLE task performance, a Student's t test was used. Results show no differences between males and females in the percentage of absolute error (PAE) of the 0–100 NLE task (*t* = −1.64; df = 90.0; p = 0.1; Cohen's d = −0.34).

We fitted five regression models to study the influence of different kinds of predictors on the 0–100 NLE performance. Specifically, we considered the influence of: (1) the control variables (Age, IQ, FAS), (2) the symbolic lexical variables (NR, ANR), (3) the Non-Symbolic semantic variables (DC, OLO), (4) the Symbolic semantic variables (ADC, ADO), and finally we tested a general model including all the best predictors from the previous four models.

In all models, the best predictors were identified using a backward selection procedure based on the Bayesian information criterion (BIC) index (Schwarz, 1978). A Bayesian approach was preferred to the traditional frequentist approach, in order to accurately estimate complex models that otherwise would fail to converge in a traditional frequentist approach (Kruschke and Liddell, 2018a). Models comparison allows for the selection of the most plausible model given data and a set of candidate models (Kruschke and Liddell, 2018b). The analyses were done using R 3.6.2 (R Core Team, 2019).

For each one of the five models, we implemented the following procedure. A full model, including all variables belonging to the specific group of predictors in analysis, has been measured through a backward selection procedure based on BIC. Specifically, using the full model as a reference, we excluded one predictor at the time, creating a set of so-called comparison models in which progressively more variables are excluded. At each step of deletion, we compared the BIC of each comparison model with the BIC of the full model by calculating the difference between the new model and the reference model (DBIC). When relevant predictors were identified, we fitted a new reference model and tried one more time to delete predictors, looking for further improvements. The best fitted model is the one with the lowest BIC, among the null, the full and the comparison models. The procedure stops when the best fit is obtained. The DBIC estimates how much the predictors affected the dependent variable. See Table 3 for details.

**Table 3:** Models' selection details: null model, full model and best fit model for each group of variables and related BIC indexes and DBIC values.

**Group of variables**	**Model**	**BIC**	**DBIC**
Age, IQ, FAS (control variables)	Null model (PAE ~ 1)	269.12	(A) −1.55
	Full model (PAE ~ AGE+IQ+FAS)	275.77	(B) −8.20
	Best fit model (PAE ~ FAS)	267.57
Number recognition (NR), Arabic Number reading (ANR), (Symbolic lexical variables)	Null model PAE ~ 1)	269.12	(A) −8.11
	Full model (PAE ~ NR+ANR)	265.47	(B) −4.46
	Best fit model (PAE ~ ANR)	261.01
Dot comparison (DC), object linear order (OLO) (Non-Symbolic semantic variables)	Null model (PAE ~ 1)	269.12	(A) 0
	Full model (PAE ~ DC + OLO)	273.84	(B) −4.72
	Best fit model (PAE ~ 1)	269.12
Arabic digit comparison (ADC) Arabic digits linear order (ADO) (Symbolic semantic variables)	Null model (PAE ~ 1)	269.12	(A) −18.66
	Full model (PAE ~ ADC + ADO)	250.46	(B) 0
	Best fit model (PAE ~ ADC + ADO)	250.46
Final Model: FAS, ANR, ADC, ADO	Null model (PAE ~ 1)	269.12	(A) −18.66
	Full model (PAE ~ FAS + ANR + ADC + ADO)	256.32	(B) −5.86
	Best fit model (PAE ~ ADC + ADO)	250.46

DBIC (A), difference between best fit model's BIC and null model's BIC; DBIC (B), difference between best fit model's BIC and full model's BIC.

The results of the first model which considered the influence of the control variables (Age, IQ, FAS) on NLE performance showed that the best model is the one in which PAE was predicted only by the FAS, although it explained only 6% of the variance of the NLE performance. The results of the second model which considered the influence of symbolic lexical variables (Number Recognition NR; Arabic Number reading, ANR) showed that the best model is the one in which PAE was predicted only by the ANR, which explained 12% of the variance in the NLE performance. The third model that considered Non-Symbolic semantic variables (Dot comparison, DC; object linear order, OLO) showed that the best model is the null one, indicating that non-symbolic tasks do not predict NLE performance. The fourth model that included Symbolic semantic variables (Arabic digit comparison, ADC and Arabic digits linear order, ADO) showed that the best model is the full model in which both ADC and ADO as predictors explained 26% of the variance in the NLE performance. The results of the fifth final model which included all the best predictors from the previous four models (FAS, Arabic number reading, ANR, Arabic digit comparison, ADC, Arabic digits linear order, ADO) showed that the best model includes both Symbolic semantic tasks (ADC and ADO), as the only predictors of the PAE and explained 26% of the variance in the NLE performance (see Table 4 for details).^1^

**Table 4:** Best fit models for each group of variables.

**Dependent variable**	**Predictors**	**β**	**Std. error**	***t*-value**	***p*-value**	**Total *R*^2^**
Control variables model—PAE 100	FAS	−0.25	0.12	−2.49	0.01	0.06
Symbolic lexical variables model—PAE 100	ANR	−0.35	0.09	−3.64	<0.001	0.12
Non-Symbolic semantic variables model—PAE 100	None	-	-	-	-	-
Symbolic semantic variables model- PAE 100	ADC	−0.37	0.09	−3.94	<0.001	0.26
	ADO	−0.26	0.09	−2.82	<0.001	
Final model—PAE 100	ADC	−0.37	0.09	−3.94	<0.001	0.26
	ADO	−0.26	0.09	−2.82	<0.001	

PAE 100, percentage of absolute error 0–100 NLT; FAS, children's socioeconomic status; ANR, arabic numbers reading; ADC, comparison of Arabic digits; ADO, Arabic digits linear order (N = 92).

## 4. General discussion

In our work, we studied the ability to represent numbers on a physical number line in 5-year-old children, with the aim of determining how NLE performance is associated with non-symbolic representations of quantities and/or with symbolic number knowledge. We tested 92 kindergarteners with the 0–100 NLE task and a set of non-symbolic tasks (dot comparison and linear object ordering) and symbolic semantic tasks (comparison of Arabic digits and linear ordering of Arabic digits). To control for the influence of numerical symbol knowledge, two lexical tasks (a number recognition task and a number reading task) were also included. To our knowledge, this work, together with van‘t Noordende et al. (2020), is the first study to investigate the predictors of the NLE task among children in this age range.

We found that young children's performance on the NLE task is predicted only by their symbolic knowledge. More specifically, our regression analysis shows that the two symbolic semantic tasks, considered together, explain 26% of the variance in the NLE performance, while non-symbolic tasks do not predict NLE performance at all. Our results are consistent with the longitudinal study by van‘t Noordende et al. (2020), who found that the dots comparison task (non-symbolic) does not predict symbolic 0–10 NLE performance.

Our results are also in line with other works that measured correlations between symbolic/non-symbolic tasks and the NLE task in older children. For example, Sasanguie et al. (2013) found a correlation between the NLE task and a symbolic comparison task but not between the NLE task and non-symbolic tasks such as magnitude comparison in 6 to 8 children. Moreover, our results support previous findings that showed that only symbolic comparison tasks, but not non-symbolic ones are associated with NLE performance in senior kindergarteners (Daker and Lyons, 2018) and first-grade children (Hawes et al., 2019).

Our data, extending the above-mentioned findings to a sample of younger children who have not yet begun formal schooling, show that NLE task processing is more based on symbolic abilities than on non-symbolic processing. Specifically, even at this young age and in the presence of an unfamiliar number range, non-symbolic tasks do not predict NLE performance. Our findings are consistent with the possibility of a separation between non-symbolic and symbolic magnitude representations already in the kindergarten age range.

Interestingly, we also found that number lexical knowledge (i.e., the ability to identify numerical symbols and to correctly read an Arabic digit) is not crucial to the NLE task, whereas semantic knowledge about the meaning of a numeral (e.g., Arabic digit comparison) is.

Therefore, although the NLE task has often been conceptualized as a good index of the representation of numerical quantities, it may be, more specifically, interpreted as a measure of symbolic quantities' representation, already in young kindergarteners. On the one hand, our results support the evidence that the NLE task is a reliable measure of symbolic numerical skills. On the other hand, our findings underline the weakness of the symbolic NLE task as a valid measure of non-symbolic numerical processing.

The primacy of symbolic over non-symbolic processing in the NLE task is consistent with the evidence of a link between the NLE task and broader mathematical skills (see Schneider et al., 2018; Bull et al., 2020 for a meta-analysis).

One implication of our results is that they may help to clarify the role of symbolic and non-symbolic numerical processing in NLE performance, and specifically whether estimation skills that come into play in this task are due to a format-independent mental representation of numerical magnitude or to the ability to access numerical magnitude through abstract symbols such as Arabic numerals.

We think that such clarification may be relevant to better understand the nature of the NLE task in its strong relationships with math skills and because of its wide use in the educational context.

Moreover, understanding the role of symbolic and non-symbolic skills in NLE performance has a practical implication for students' future learning in educational settings. Considering that the NLE task is frequently used as a measure of math skills in school and pre-school contexts, the knowledge of its predictors (symbolic or non-symbolic) supports the choice of which basic math skills to enhance already at the preschool level. In particular, our findings suggest that, in order to optimize the development of numerical understanding in kindergarteners, schools should implement a stronger emphasis on children's understanding of the symbolic system over their analogical magnitude reasoning.

From a theoretical point of view, our results provide new elements to the symbolic vs. non-symbolic debate on early math cognitive development. Extending the results of Xenidou-Dervou et al. (2015) who identified different developmental trajectories for symbolic and non-symbolic magnitude representations as early as first grade, our data suggest that this divergent path could start even before primary school.

## 5. Conclusions

The present study focuses on the emergence of symbolic knowledge of numbers in kindergartners, which is an area of cognitive numerical development that has not been sufficiently investigated in the past. Our results indicate that symbolic knowledge about quantities and numerals may play a stronger-than-expected role in the stage of development in which children are not yet very familiar with culturally determined numeric symbols.

A limit of our study is the lack of longitudinal control of the development of symbolic and non-symbolic skills. A follow-up study can address this limitation by longitudinally testing the same sample at different stages of cognitive development. Another limitation is that the list of numbers used in the NLE task are not equally distributed before and after the midline point. Future research directions should include the comparison of symbolic and non-symbolic abilities using both symbolic and non-symbolic versions of the NLE task, in order to observe the effects of symbolic and non-symbolic predictors involved; furthermore, number lines could be used with different ranges, more or less familiar to the child, and balanced target lists in which numbers smaller and bigger than “50” are equally represented.

Further investigation should be conducted to better clarify crucial aspects of numerical development (e.g., Siegler, 2016) such as how symbolic numbers are connected to their non-symbolic referents and how accurate is the representation of the magnitude of rational numbers at these early stages of numerical cognitive development.

## Data availability statement

The raw data supporting the conclusions of this article will be made available by the authors, without undue reservation.

## Ethics statement

The studies involving human participants were reviewed and approved by Department of Pedagogy, Psychology and Philosophy, University of Cagliari Ethics Committee. Written informed consent to participate in this study was provided by the participants' legal guardian/next of kin.

## Author contributions

CM and RF were involved in the planning of the study and supervised the collection of data. CM did the data analysis, wrote the introduction, method and results sections. RF wrote the general discussion and conclusions sections of the manuscript and FD revised the entire manuscript. All authors contributed to the article and approved the submitted version.

## Footnotes

^1^We also run a hierarchical regression analysis, with all variables including gender. In the first step, we included all the control variables, in a second step the symbolic lexical variables, in a third step the non-symbolic semantic variables and in the last step the symbolic semantic variables. The analysis yielded the same results as the Bayesian analysis.
